# Developing a Culturally and Linguistically Congruent Digital Storytelling Intervention in Vietnamese and Korean American Mothers of Human Papillomavirus–Vaccinated Children: Feasibility and Acceptability Study

**DOI:** 10.2196/45696

**Published:** 2023-06-14

**Authors:** Sunny Wonsun Kim, Angela Chia-Chen Chen, Lihong Ou, Linda Larkey, Michael Todd, Yooro Han

**Affiliations:** 1 Edson College of Nursing and Health Innovation Arizona State University Phoenix, AZ United States

**Keywords:** Vietnamese, Korean, Asia, cultural, digital storytelling, storytelling, story, stories, HPV, vaccine, vaccination, feasibility, digital intervention, mortality rate, ratio, odd, rate, deep analysis, social media, child, immigrant, mother, immunization, inoculation, inoculate, communication, culture, language, human papillomavirus, photo, video, digital, microphone, conversation, dialogue, Research Electronic Data Capture, voiceover, soundtrack, writing, write, script, health status, health insurance, survey, questionnaire, qualitative, constructivist, constructivism

## Abstract

**Background:**

The high morbidity, mortality, and economic burden attributed to cancer-causing human papillomavirus (HPV) call for researchers to address this public health concern through HPV vaccination. Disparities of HPV-associated cancers in Vietnamese and Korean Americans exist, yet their vaccination rates remain low. Evidence points to the importance of developing culturally and linguistically congruent interventions to improve their HPV vaccination rates. We adopted digital storytelling (DST) that combines oral storytelling with computer-based technology (digital images, audio recording, and music) as a promising approach for facilitating the communication of culturally relevant health messages.

**Objective:**

This study aimed to (1) assess the feasibility and acceptability of intervention development through DST workshops, (2) conduct an in-depth analysis of the cultural experience that shapes HPV attitudes, and (3) explore aspects of the DST workshop experience that could inform future formative and intervention work.

**Methods:**

Through community partners, social media, and snowball sampling, we recruited 2 Vietnamese American and 6 Korean American mothers (mean age 41.4, SD 5.8 years) who had children vaccinated against HPV. Three virtual DST workshops were conducted between July 2021 and January 2022. Our team supported mothers to develop their own stories. Mothers completed web-based surveys before and after the workshop and provided feedback on each other’s story ideas and the workshop experience. We used descriptive statistics to summarize quantitative data and constant comparative analysis to analyze qualitative data collected in the workshop and field notes.

**Results:**

Eight digital stories were developed in the DST workshops. They were well accepted, and the mothers showed overall satisfaction and relevant indicators (eg, would recommend it to others, would attend a similar workshop, it was worth their time; mean 4.2-5, range 1-5). Mothers found the process rewarding and appreciated the opportunity to share their stories in group settings and learn from each other. The 6 major themes that emerged from the data reflect the mothers’ rich personal experiences, attitudes, and perceptions about their child’s HPV vaccination, which included (1) showing parents’ love and responsibility; (2) HPV and related knowledge, awareness, and attitudes; (3) factors influencing vaccine decision-making; (4) source of information and information sharing; (5) response to children's being vaccinated; and (6) cultural perspectives on health care and HPV vaccination.

**Conclusions:**

Our findings suggest that a virtual DST workshop is a highly feasible and acceptable approach to engaging Vietnamese American and Korean American immigrant mothers in developing culturally and linguistically congruent DST interventions. Further research is needed to test the efficacy and effectiveness of digital stories as an intervention for Vietnamese American and Korean American mothers of unvaccinated children. This process of developing an easy-to-deliver, culturally and linguistically aligned, and holistic web-based DST intervention can be implemented with other populations in other languages.

## Introduction

The human papillomavirus (HPV) is the most common sexually transmitted infection in the United States, with more than 42 million Americans being infected with types that cause diseases in 2022. This number is projected to increase by 13 million new infections each year [1]. HPV infection is strongly associated with cancers in both sexes, such as cervical cancer in females, penile cancer in males, and oropharyngeal cancers in both sexes [2]. Specifically, HPV types 16 and 18 were found to be linked to nearly 70% of cervical or oropharyngeal cancers in the United States, and these 2 types of cancers were identified as the most common HPV-related cancer in females and males, respectively [3,4]. It was projected that 14,100 Americans would receive a diagnosis of cervical cancer and 54,000 would be diagnosed with oral cavity or oropharyngeal cancer in 2022 [1,2]. HPV vaccines offer a promising approach to providing safe, effective, long-lasting protection against HPV-related infections and associated cancers [5]. Vigorous prevention efforts including HPV vaccination for boys and girls at age 11 or 12 years are recommended by the Centers for Disease Control and Prevention [1,6], but uptake remains low in many population groups.

Data on HPV-associated cancers or vaccination rates in Asian American men are lacking, but Asian American women are known to be disproportionately affected by HPV-associated cervical cancer. Despite the higher risk among Asian American women, their vaccination rates remain low. Among Asian American subgroups, Vietnamese American (18.9 per 100,000) and Korean American (11.9 per 100,000) females had the highest rates of cervical cancer mortality compared with White females (7.1 per 100,000) [7]. Although the proportion of those dying from cervical cancer did not differ between Asian American and White women, Korean American women diagnosed with cervical cancer had worse overall survival compared with White women [8]. Troublingly, the HPV vaccine completion rate is only 9% for Vietnamese American women [9] and 33.3% for Korean American women [10]. Additionally, the HPV vaccine coverage with more than 1 dose of the vaccine was also found to be low among Asian American adolescents aged 13-15 years, with estimated completion rates of 60% for girls and 41% for boys [11], substantially below the Healthy People 2030 goal of 80% vaccine coverage of adolescents [12].

Given the need for parental consent, and the unique context of mothers’ health-related decision-making in Asian cultures [13,14], it is crucial to engage Vietnamese American and Korean American mothers in HPV vaccination. In this paper, culture is defined as the system of rules, meaning, and beliefs shared by a large group of people, and more broadly, in how the conversations based on that code in the community guide health decisions, all of which can be represented in shared narratives of a cultural group [15]. Mothers play an important role in HPV vaccination uptake in their children, with mothers as the primary attendee at doctors’ visits, particularly in Asian populations [14]. Factors such as mothers’ limited English proficiency, lack of knowledge about the HPV vaccine, or beliefs that vaccination would encourage children’s premarital sex contribute strongly to the lower rates of HPV vaccination in the Vietnamese American and Korean American populations [9,10]. Kim et al [16] found that informing Korean American mothers about HPV can successfully increase their HPV knowledge. These findings underscore the importance of developing culturally and linguistically congruent interventions to improve HPV vaccine uptake in Vietnamese American and Korean American, particularly among first-generation immigrants (individuals born outside of the United States) [17]. While Vietnamese American and Korean American mothers’ attitudes, beliefs, and intentions to have their children vaccinated are keys for promoting HPV vaccination [9,18], there is a paucity of rigorous research engaging Vietnamese American and Korean American mothers in the process of developing culturally and linguistically congruent interventions. Furthermore, only limited research has focused on both boys and girls, despite vaccination proving to be effective in preventing HPV-associated cancers in both sexes [5].

Storytelling, a specific form of cultural narrative, shows promise as an effective culture-centric health promotion strategy [15,19]. Digital storytelling (DST), which combines oral storytelling with computer technology, has been used as a tool to communicate culturally relevant messages in health, education, and community settings [20]. The process of DST, an innovative community-based participatory research method, involves the production of individuals’ own brief visual stories presented in their own voice, incorporating photographic images, music, and artwork of their choice in a workshop setting guided by professionals and is 1-3 minutes in length [20]. DST embraces a person-centered approach and incorporates community-based participatory research methods that allow marginalized community members to retain control over their own stories and collectively make sense of lived experiences [21].

Research examining DST interventions has demonstrated its potential benefits for promoting different health behaviors, including HPV vaccination among youth and adult participants [18,22]. However, to our knowledge, other than our pilot study developed with 2 Vietnamese American mothers [22], no DST intervention has been developed to address HPV vaccination targeting either Vietnamese American or Korean American immigrant mothers. In addition, little is known about how the process of creating and sharing a personal narrative may be linked to their unique cultural view of HPV attitudes and behavior to promote vaccination. To address the gaps, we sought to explore the feasibility and acceptability of developing an intervention through the DST workshop process in a group of Vietnamese American and Korean American mothers of vaccinated children against HPV recruited from the community. Our team conducted 2-day web-based DST workshops to assess (1) feasibility; (2) acceptability; (3) in-depth analysis of mother’s cultural experience that shapes HPV attitudes and behavior, and perceptions about their child’s HPV vaccination; and (4) exploration of the experience of the DST workshop that could shape future work.

## Methods

### Participant Recruitment and Samples

Adult women (18 years or older) who (1) self-identified as Vietnamese American or Korean American; (2) were first-generation immigrants born outside of the United States; (3) were mothers or female primary caregivers with one or more boys or girls aged 11-14 years old who had completed the recommended doses of the HPV vaccine; and (4) spoke English, Korean, or Vietnamese were invited to participate in this study. English fluency was not required, given the bilingual resources of the research team. If an eligible mother had more than one vaccinated child aged 11-14 years old, we asked them to answer the eligibility questions based on the oldest child. Exclusion criteria included inability to adhere to the study protocol, which included attendance at a 2-day virtual workshop. Initial screening was conducted via a brief phone or in-person interview. Potential participants were screened for eligibility, and full informed consent was obtained from all the eligible participants. With the help of bilingual or bicultural community navigators from the Asian Pacific Community in Action and The Arizona Partnership for Immunization, we recruited participants from the greater Phoenix metropolitan area via word of mouth; community-based organizations; clinics; social media; and Vietnamese and Korean language radio broadcasts, newspapers, and flyers posted in local Asian supermarkets, churches, and nail salons between December 2021 and January 2022. Each participant received monetary incentives upon successful completion of the 2-day workshop.

### Ethics Approval

The study was approved by the Institutional Review Board (IRB: STUDY00011207 and STUDY00011733) at Arizona State University, and all materials were administered in accordance with relevant guidelines and regulations and translated and back-translated to Vietnamese and Korean using institutional review board protocols. Informed consent was obtained from all participants. The confidential nature of material produced during the workshop (eg, story scripts, digital images, transcripts from group discussions) was emphasized to participants: they had the option to remain anonymous within their stories by omitting names and dates and having images blurred. Participants were informed about our future aim to disseminate the digital stories for research purposes.

### Study Design

#### The DST Workshop Overview

The DST workshop format was designed by the StoryCenter, a California-based organization considered to be the founders of the DST movement. DST workshops are most easily and effectively facilitated over two to three 8-hour sessions conducted on consecutive days. DST comprises three components: (1) an individual process; (2) a group process; and (3) a process co-mediated by participants, researchers, and facilitators [21]. Each attendee shares a story verbally, in a group setting called a “Story Circle”; records the story into a microphone; chooses or creates photos and video clips to illustrate the story; and works with the workshop facilitators to combine these materials into short digital videos called “digital stories.” In this activity, each participant introduces and “screens” their final personal digital story to the group, with facilitators guiding a group discussion around the content of each individual story and then the entire collection of stories produced in that particular workshop [21]. Our DST workshops were designed to have these personal digital stories created over the course of 2 consecutive 6-8–hour days.

#### Study Preparation

Prior to recruiting participants, the lead investigators discussed the DST process with the research team and developed a set of story prompts to gain an understanding of Vietnamese American and Korean American mothers’ experiences with their children’s HPV vaccinations. After participants were recruited, but prior to the workshop, they were told to think about the meaningful stories that they would want to share with the group during the workshop and to bring their own photographic images to be included in their digital videos. The lead investigators also contacted participants via email and phone prior to the workshop to provide support with gathering materials and to answer any questions.

#### Study Procedures

Due to COVID-19 safety concerns and related regulations, we conducted the virtual DST workshops via Zoom and collected web-based data via Research Electronic Data Capture (Vanderbilt University), a secure data collection and management platform [23]. Three separate 2-day workshops were conducted. The DST workshop for Vietnamese American mothers was held on 2 consecutive days in July 2021, and 2 separate 2-day DST workshops for Korean American mothers were held in December 2021 and January 2022. A team of 4 researchers served as facilitators to assist with the workshop and support participants’ sharing and learning in the workshop and practicing digital editing.

The first day of each workshop began with a 2-hour orientation session to review the DST process. Workshop participants were invited to share stories from their own life experiences, in the context of a group production environment (story circle). Three example digital stories and prompt questions were shared with the participants to help them brainstorm story ideas. The prompts (eg, “What is the story of your learning about HPV?” and “What led you to have your child vaccinated?”) were developed based on a prior workshop conducted with Vietnamese American mothers [22]. Additionally, the participants provided feedback on each other’s story ideas, the workshop experience, and the experience of working in a group. Prompt questions (eg, “How were your overall experiences, do you think that your time was worthwhile?” and “What did it really mean to you to share your story out loud in a group?”) were also used to guide the workshop evaluation. After each participant shared their HPV vaccination story, they received feedback from other participants and facilitators to elicit the most powerful story. Participants were also tasked with bringing pictures to the next day’s session to help develop their stories.

On the second day, facilitators worked with individuals to find a critical moment in their experiences and gained a sense of what the important stories might be. Then, with facilitators, each participant developed a 300-word story script and recorded the voiceover. Before recording voiceovers, participants were encouraged to practice and read their scripts out loud until they felt confident enough to be recorded. Facilitators talked participants through the steps they needed to take to complete specific editing and production tasks. A media producer helped with editing the stories by combining written and oral narratives, images, voiceover, and a soundtrack. The collaborative teamwork ensured that the final product was an authentic expression of participants’ stories. The stories were probed and shaped through this facilitation to produce narratives of meaningful individual experiences about HPV and vaccination. Facilitator teams prepared draft versions of the individual digital stories during the workshops.

At the end of each workshop, participants watched their final draft stories together and discussed aspects of each story and overall workshop experiences. Upon completion of the DST workshop, participants were asked to complete a web-based end-of-workshop evaluation, which included a satisfaction survey and open-ended questions to share their experiences with the workshop. The participants received DVDs with their own digital stories with English and Vietnamese or Korean subtitles approximately 1 month after the workshop.

### Measures and Data Collection

#### Demographics

Before the workshop, participants were asked to complete a questionnaire with items assessing age, gender, race and ethnicity, marital status, educational level, and employment status. This questionnaire also included items on self-reported health status, health insurance, family history of cancer, and number of children aged 11-14 years old in the family (Table 1).

**Table 1:** Background characteristics of study participants.

Variable		Korean American (n=6)	Vietnamese American (n=2)	Total (N=8)
Age of target child (years), mean (SD)^a,b^		12.6 (1.8)	14.0 (2.4)	13.3 (2.1)
**Sex of the target child, n (%)**				
	Male	3 (50)	0 (0)	3 (37)
	Female	3 (50)	2 (100)	5 (62)
**Education level, n (%)**				
	Bachelor’s degree	4 (67)	0 (0)	4 (50)
	Graduate degree	2 (33)	2 (100)	4 (50)
**Primary language spoken with children, n (%)**				
	Korean or Vietnamese	5 (83)	0 (0)	5 (62)
	English	1 (17)	2 (100)	3 (37)
**Health insurance, n (%)**				
	Yes	5 (83)	2 (100)	7 (87)
	No	1 (17)	0 (0)	1 (12)
**Employment status, n (%)**				
	Yes	3 (50)	2 (100)	5 (62)
	No	3 (50)	0 (0)	3 (37)
**Family history of cancer, n (%)**				
	Yes	4 (67)	2 (100)	6 (75)
	No	2 (33)	0 (0)	2 (25)
**Mother’s history of cancer** **, n (%)**				
	Yes	1 (17)	0 (0)	1 (12)
	No	5 (83)	2 (100)	7 (87)

^a^Target child was defined as the oldest unvaccinated child aged 11-14 years in the family.

^b^Range: 11-14 years.

### Data Analysis

#### Feasibility

Our first objective was to assess feasibility using recruitment, retention, and survey completion rates and direct feedback from participants from group discussions following the DST workshop. Benchmark criteria for feasibility metrics were set in accordance with the goals of a future study that will test the intervention in a larger population. We aimed to recruit an average of 2 to 4 participants per workshop, with a 60% consent rate being our benchmark. We assessed retention rate as the proportion of participants who completed a 2-day workshop and all assessments (baseline and postworkshop), with an 80% retention rate being our benchmark.

#### Acceptability

We assessed acceptability using participants’ responses to a 25-item satisfaction and workshop experience questionnaire administered at the end of each workshop. The questionnaire comprised items assessing overall satisfaction with the workshop, perceived appropriateness of the workshop content and pace, willingness to attend a similar workshop, and willingness to recommend this workshop to others. The responses were rated on a 5-point Likert scale ranging from 1 (strongly disagree) to 5 (strongly agree). Participants’ suggestions for improving the workshop were collected using yes or no items. We further assessed acceptability and satisfaction using open-ended questions about which aspects of the workshop participants found most valuable, investigator field notes, qualitative data from transcripts of story circles, story screening, and recordings of follow-up debriefing group discussions.

The web-based workshop activities were digitally recorded, transcribed verbatim, and translated into Vietnamese or Korean before analysis. Field notes were also made during the workshop to add rich descriptions of the group environment and interactions. The transcription and translation were verified for accuracy by 2 research team members and one of the lead investigators. The data were organized and analyzed using NVivo software (Release 1.6.1; QSR International) [24].

#### HPV Experiences

To meet our last 2 objectives, we are using a constructivist grounded theory approach [25], which is particularly suitable for capturing different aspects of the DST workshop based on the conceptual model of storytelling as cultural-centric health promotion [18]. The qualitative analytical process included a constant comparative analysis, line-by-line coding, and codebook development deriving from participants’ narratives and responses to each question prompt [26]. Three researchers independently reviewed transcripts of story circle, story screening, and follow-up debrief focus group recordings; field notes; and participant responses to the workshop evaluation. The emerging themes for the codes and subcodes were discussed among the research team, and exemplar quotes from participants were also added to support the identified themes. Emergent themes were then compared across all groups until the intercoder reliability was consistently at least 80% on 95% of the codes [27]. Through this process, 3 researchers independently identified the codes, themes, and discrepancies in the analytic results that were discussed and resolved until a consensus was reached. In this paper, we present a triangulation of findings from the data to qualitatively analyze the DST process. All quantitative analyses were conducted using SPSS statistics (version 27.0; IBM Corp) [28]. Descriptive statistics were used to describe the demographic characteristics and summarize the survey data.

## Results

### Sample Description

The sample comprised 8 female participants (mean age 41.4, SD 5.8 years) who had children vaccinated against HPV. Among them, 2 were Vietnamese mothers and 6 were Korean mothers. All mothers were immigrants (born outside of the United States). Seven out of 8 had health insurance, and their children were not eligible for free or reduced lunch at school. Four out of 8 were working full-time and reported family cancer history. Five were fluent in English. Two Vietnamese mothers participated in the DST workshop in July 2021, 4 Korean mothers participated in December 2021, and another 2 Korean mothers participated in January 2022.

### Objective 1: Feasibility

A total of 14 prospective participants agreed to be contacted and were approached. Of those, 3 people declined to take part because of a schedule conflict, and 3 were ineligible due to their children’s HPV vaccination status. Eight (73%) consented to participate in the study, all of whom (100%) attended the entire 2-day DST workshop, completed pre- and post-workshop surveys, and successfully created their own digital stories embedded with self-selected sound and images or photos. Each video was around 3-5 minutes long. Although the number of Vietnamese mothers (n=2) in the workshop was lower than the target goal, the overall study recruitment and retention rates exceeded our benchmark values of 60% and 80%, respectively.

### Objective 2: Acceptability

In general, the DST workshops were well accepted and liked among both Vietnamese American and Korean American mothers (Table 2). Using 5-point rating scales, participants reported high satisfaction with the workshop (mean 5.0, SD 0.0), indicating that they would recommend it to others (mean 4.4, SD 0.37), would like to attend a similar workshop (mean 4.6, SD 0.59), the workshop was worth their time (mean 4.7, SD 0.55), it was appropriate for them (mean 4.7, SD 0.55), and it lived up to their expectations (mean 4.2, SD 1.10). A majority of participants commented that they liked the pace; communication about the workshop purpose; the total amount of the content and quality of the content; the methods, pace, flow, and difficulty level of the workshop; the activities it included; and the number of workshop attendees. Open-ended responses offered a broader and deeper perspective on the acceptability of DST. These responses were categorized in terms of acceptability of DST purpose and acceptability of DST format. Participants expressed overall satisfaction with the purpose of DST, noting that attending the workshop was beneficial and that it was particularly important to share their own stories in group settings and learn from each other.

**Table 2:** Participants’ ratings of acceptability with the workshop (N=8).

Satisfaction item^a^	Korean American, mean (SD)	Vietnamese American, mean (SD)	Total, mean (SD)
What is your overall satisfaction with the workshop?	5.00 (0.00)	5.00 (0.00)	5.00 (0.00)
To what extent was attending this workshop worth your time?	4.83 (0.38)	4.50 (0.71)	4.67 (0.55)
The workshop was appropriate for me	4.83 (0.38)	4.50 (0.71)	4.67 (0.55)
This workshop lived up to my expectations	4.33 (1.10)	4.00 (0.00)	4.20 (1.10)
Would you attend a similar workshop again?	4.67 (0.47)	4.50 (0.71)	4.59 (0.59)
I would recommend this workshop to others	4.83 (0.37)	4.00 (0.00)	4.42 (0.37)

^a^Scale range: 1-5 for all items.

### Objective 3: Overall Experiences of the Workshop

During the story screening phase, mothers shared their experiences of the DST workshop, the group environment, and thoughts about the group size and workshop schedules. Although sharing one’s story in a group could be stressful, they found it rewarding. They all expressed gratitude and satisfaction with the workshop:

It has been quite invaluable.

I’ve never done on storytelling before, so it's practically my first time doing this...I really found it exciting.

The mothers also appreciated the engaging learning experience that the group environment fostered. They found that personal stories are meaningful as a health promotion tool to increase awareness of vaccination at the community and societal levels. In addition, participants reported that they could relate to each other’s stories as first-generation immigrant mothers.

I was so compassionate, and they made me felt for her

another study participant

We are all moms, and we'll have it all figured out and I guess we learn along the way, and that's really great.

The participants also felt satisfied with the workshop duration and the group size, and appreciated being able to participate in the digital workshop. Although 1 mother expressed concerns about time constraints on writing her story script, she still expressed satisfaction with the workshop schedule.

The participants found the digital approach beneficial in terms of convenience and the broad impact beyond geographical boundaries:

Really be convenient, I really even prefer the zoom section.

Even if you want to hold an impact in person section, you have to do it with people within your reach to what with the aid of social media with the aid of zoom, you can reach out to people for different session and another benefit of this is that you can do it from the comfort of your home.

Regarding the group size, mothers recognized the pros and cons of small versus large groups, but they all preferred smaller groups due to time constraints:

I think the smaller for me it's better, if it is larger, I think it creates much more time than smaller size.

### Objective 4: A Cultural View of the HPV Vaccination: Emergent Themes Drawn From DST Narrative Data

#### Mothers’ Attitudes and Perceptions Regarding Their Child’s HPV Vaccination

Six major themes reflecting the mothers’ rich personal experiences, attitudes, and perceptions about their child HPV vaccination emerged from the data. These themes were: (1) showing parents’ love and responsibility; (2) HPV and related knowledge, awareness, and attitudes; (3) factors influencing vaccine decision-making; (4) sources of information and information sharing; (5) responses to their children being vaccinated; and (6) cultural perspectives on health care and HPV vaccination. Overall experiences of the workshop and additional thoughts and suggestions were also collected from the participants during the postworkshop discussion sessions.

#### Showing Parents’ Love and Responsibility

All participants (n=8, 100%) addressed the power of parental love and a sense of responsibility to protect the family and provide the best for their children. Specifically, the role of a mother in children’s health and caring for their future health was highlighted as one of the key driving factors for parents to vaccinate their children.

#### The Role of a Mother as a Caregiver for Their Children

The significant role a mother played in children’s lives and health was evident in the participants’ quotes.

I made a plan for myself, and I said I’m going to give my kids the best I could give to them.

After raising a baby and becoming a mother, I started to think a little more about vaccines.

Planning ahead and preparing for vaccinations even before a child was born became necessary for mothers to ensure a healthy start in children’s life.

Even before my child was born, I had a list of vaccines.

#### Giving Priority to Caring for Family’s Future Health

The mothers valued the vaccine decision-making as a contribution to children’s future health.

The moment I chose to vaccinate my child was the moment I made the first decision for my child…I chose for the future of my child to protect my son and to protect his future family, too.

Another common consideration to vaccinate a child was protecting them and others. One mother also described that using vaccines was a simple way to safeguard children’s health and well-being.

If I can help prevent any type of diseases in my boys, I will do it... the easiest thing that you can do is to protect your kids by getting them fully vaccinated.

#### HPV and Related Knowledge, Awareness, and Attitudes

Two subthemes emerged that reflected mothers’ knowledge, awareness, and attitudes about HPV and HPV vaccinations: lack of knowledge and awareness about HPV vaccination, and positive attitudes toward HPV and HPV vaccinations.

#### Lack of Knowledge and Awareness About HPV Vaccination

Although the participants demonstrated some knowledge and awareness about the HPV vaccine, some of them discussed having first heard about the disease, confusing it with the HIV, or being educated about it only as a female-specific disease. The mothers said:

I didn't know much about vaccines and viruses.

When I was studying that virus, I was basically educated as a woman's disease.

One participant also reflected on her mother’s lack of important knowledge on the HPV vaccine and how that led to missed opportunities to best protect her sister who was later infected with the virus:

We pride ourselves on being a well-educated family….I was surprised that my mother didn't know anything about HPV and that she didn't protect my sister who then later was contracted with the virus.

#### Positive Attitudes Toward the HPV Vaccines

The participants generally had positive attitudes toward the vaccine, and they expressed no doubts about the value of HPV vaccination in protecting individuals, the family, and the community and thought it was necessary.

Vaccinations that protect everyone and that's the goal we want.

If it can protect against cancer, I don't think there is anyone who wouldn't get vaccinated for both boys and girls.

To ensure timely vaccination coverage for her child, 1 mother approached the medical assistant and asked whether her daughter was eligible for the HPV vaccination. She advocated for her child's health care, which could benefit the child's future as well.

When I took my daughter for her eleventh or ten-to-eleven-year checkup and I remember…asking the immunization shots for this year.

#### Factors Influencing Vaccine Decision-Making

##### Overview

Five factors behind the participants’ vaccine decision-making were found in the workshops: (1) lack of provider communication about HPV and vaccination, (2) family members’ or friends' experience or perspectives, (3) weighing the benefits and risks before making the decision, (4) concerns about the timing of the vaccination, and (5) increasing skepticism toward vaccination.

##### Lack of Provider Communication About HPV and Vaccination

Receiving a reluctant response from the health care provider when reaching out to them for advice regarding the HPV vaccine could place parents in a difficult position. One participant described that the medical assistant seemed surprised when she tried to seek advice from her on vaccinating her child against the HPV. She said:

I was kind of disappointed that my own pediatrician and staff didn't even bother to talk to me about protecting my child against cervical cancer in the future and this virus that could stay in the system for the rest of their lives.

##### Family Members’ or Friends' Experience or Perspectives

The health outcomes of unvaccinated people close to the mothers could influence their decisions to vaccinate against HPV. One mother discussed the costs of being unvaccinated and shared a sad story of her friend’s child. The potential suffering from the disease and its consequences as a result of lacking effective protection that a vaccine could offer had a great impact on her vaccine acceptance. The previous experience of family members also demonstrated a similar impact. Another participant discussed her feeling of regret for not being there with her mother and sister to support their vaccine decision-making:

I was at fault because I wasn't there...to kind of nudge my mother

to have my younger sister get vaccinated

##### Weighing Benefits and Risks Before Making a Decision

The mothers also discussed how the information they gathered informed them about both the benefits and risks of the vaccination. When the perceived benefits were outweighed by perceived risks, they were much more likely to decide to vaccinate their children:

I could feel upset about my wrong decision, but I came to the conclusion that there were more positive things than that, and I changed my mind that I should vaccinate

my child

##### Concerns About the Timing of Vaccination

As parents usually jointly decided their children’s vaccination, they might have different perspectives concerning the needs and timing of the HPV vaccination. One mother noted that she had considered the HPV vaccine but the biggest concern that she discussed with her husband centered on the best time for their child to receive the vaccine.

##### Increasing Skepticism Toward Vaccination

Despite mothers’ general belief in the value of the HPV vaccine, they also felt hesitant about vaccinating themselves and having their children vaccinated. Fear of needles and concerns about the side effects of the vaccine were the 2 primary factors that were associated with vaccine hesitancy. One mother expressed her extreme fear of injections:

I am not an injection kind of person, even if I’m really sick to the point of me dying I still reject injections.

The mother participants appeared to take a more cautious approach when making the decision on vaccinating their children, especially when facing information overload. As described by the participants:

I was positive about vaccination, but as the child grew up, I was shaken in an environment where there was a lot of information.

The information related to any adverse events and side effects from the vaccine could affect mothers’ willingness to vaccinate their children.

I came across a lot of side effects through YouTube or the Internet, I changed my mind a bit negatively.

### Source of Information and Information Sharing

#### Seeking Credible Sources of Information

Health care providers, radio news, and web-based information were identified as the primary sources of vaccine information. The majority of the participants discussed the role of health care providers (eg, pediatricians) in their learning about HPV and the vaccine. The health care providers specifically offered advice on the eligible age for the vaccine and the benefits of the vaccine. They also shared paper-based learning materials with the mothers and explained how vaccination worked. Some mothers sought additional source information from the internet, and radio channels, such as Google, YouTube, National Public Radio, Reuters, and Associated Press News.

Sometimes [I] go to the Internet and get some information.

I talked to the doctor and after talking to the doctor, I also went to the Internet browsers one or two devices to see.

In addition, 1 mother reported that she and her daughter shared the plan of vaccination with their friends. The variety of sources that the participants had access to contribute to their awareness and knowledge of the HPV vaccine, and further supported their vaccine decision-making and behavior.

But sometimes health care providers did not recommend the HPV vaccine as they did for other routinely recommended vaccinations. Not initiating conversations around HPV preventive measures and recommending the vaccine to parents could be critical barriers to increasing parents’ knowledge and awareness of the vaccine. The lack of information kept parents from appropriately assessing the benefits and risks for their children to receive HPV vaccinations. One mother said, “I wish my doctor gave me enough information to be decisive, especially for my boys.”

#### Concerns About Misinformation

Despite the availability of multiple sources of information, some mothers expressed concerns about the prevalence of rumors and misinformation surrounding the HPV vaccine on social media platforms. When realizing the significance of trusted sources in driving their vaccine decision-making, the mothers strived for finding the information coming from credible sources. One participant said:

When I see things online, I make sure that I’m getting it from sources that are reliable. So, I tend to kind of collect a lot of data ...so that I can confirm what I’m reading what I’m seeing.

Vaccine-related information from noncredible sources could increase confusion and cause unnecessary anxiety among parents. The trust in health care providers and science and seeing them as credible sources of information also had a great impact on mothers’ willingness to vaccinate their children against HPV. They stated:

There's no question about the science or anything like that.

I had to like talk to my doctor…I took that leap of faith and I really trust him.

#### Response to Children’s Being Vaccinated

The participants prompted similar responses when sharing feelings upon completion of their children’s HPV vaccination:

I made the choice and vaccinated my three boys. I made a good decision.

My joy was restored.

One participant also described that she was surprised to find her daughter happy about being vaccinated and the receipt of the vaccination made her feel proud of her daughter:

She was so happy to be vaccinated, and she couldn't wait for the day to come… this made me I was so proud of her.

Nevertheless, there could be some complexities of vaccine hesitancy and acceptance in the family at the same time. It happened to one mother that her husband vaccinated the child without her consent and awareness:

When I heard this story, my child’s checkup was approaching and, in a situation, where I was worrying about the vaccination. When my husband and 14-year-old returned, she had already gotten vaccinated.

### Cultural Perspectives on Health Care and HPV Vaccination

#### Cultural Aspects

Cultural aspects also played a role in determining the mothers’ perspectives on adolescents’ sexual health and the decision-making related to vaccinating their children. Mothers did not feel comfortable talking about sexuality, as they seemed to view it as a taboo topic for discussion. One participant said:

The subject of STD, HPV was never brought up in my family, my dad took me out of sex-ed in elementary school.

This perspective could lead to low awareness of preventive sexual health measures generally and delays in HPV vaccination specifically. One mother said:

When I asked the doctor when would be a good time and heard that it is good for my child to be vaccinated before having sex, as a conservative Asian mother’s point of view, I thought it was still a long way.

Participants also highlighted that women's health was often neglected in their cultural context and that there had been little or no attention paid to preventive health initiatives for women:

Female health is not something that they paid attention to.

Another participant made the decision to have herself vaccinated against HPV when she was a young adult without involving her father, she said:

I got the HPV vaccine against my dad's wishes; I actually did it without even asking him.

Taking an active role in seeking different sources of credible information can be helpful in advocating for oneself and in making less biased health care decisions, but exercising this kind of autonomy by questioning professionals’ opinions or practices can be a challenge for Asian mothers or parents. As one mother described:

I think one of the things that we [Asians] have in common is that we revered those who are educated, those who are professionals and teachers, doctors, and we put them up on a pedestal and we think, oh well they can't be wrong, right, so everything they say has to be right. But then, as I interact more and more of them, and I realize well no they can be wrong, and I need to speak for myself because I know my body, I’ve been living in this body for however many years, so I know it. And so, I need to ask the questions of different people.

#### Navigating Different Health Care Systems in the United States

When reflecting upon the cultural differences between the United States and Asia and their impacts on the health care practice and vaccine decision-making, mothers reported similar vaccine practices despite the different preferences in treatment options between the 2 systems:

I thought that the doctor suggested vaccination because it was the United States, which has a sexual life and an open environment, but unexpectedly, the children of many doctor families in Korea I know were also vaccinated.

One mother also considered the use of advanced resources in the US health care system as a privilege:

No matter what our traditions are what our values may have been, but the advances that have been made by other people for our benefit we need to take advantage of that.

Some participants explained that as first-generation immigrants they had experienced some confusion and frustration with the US health care system and had difficulties using or accessing health care services. Although they had health insurance, they knew that health care costs were significantly higher in the United States than in Korea or Vietnam.

The U.S. health system has been changing a lot, but there is a lack of information for Korean Americans. I wish we had some types of health information about the health system or insurance in Korean. Thus, it is very helpful to ask my other Korean friends and share some information.

## Discussion

### Major Findings

In this study, we assessed the feasibility and acceptability of developing a DST intervention and explored a deeper understanding of Vietnamese American and Korean American mothers’ rich personal experiences, meaning-making, attitudes, perceptions, and cultural views regarding their child HPV vaccination during three 2-day group-based DST workshops among Vietnamese American and Korean American immigrant mothers with children vaccinated against HPV. Although prior studies have examined the feasibility and acceptability of DST and its impact across diverse patient populations (cancer survivors, diabetic patients, patients experiencing stress) [29-34] and across different racial and ethnic groups (African American, Indigenous, and Latina women) previous to our own work, no DST studies with Vietnamese and Korean immigrants had been conducted, and no DST interventions had been designed to address the persistent HPV vaccination disparities among children of Vietnamese American and Korean American immigrant mothers. Gubrium et al [21] and Drenkard et al [35] discussed the challenges they faced in recruiting and retaining participants for workshops offered over consecutive days, given that disruptive life events and work schedules were limitations to minority participants’ engagement in particular. The 100% retention rate for our 2-day virtual DST workshops suggests that the digital format used in the current study was effective for retaining participants. Satisfaction survey responses and qualitative data showed that our participants had high overall satisfaction with the DST workshops, the experience of story sharing in a group environment, the feeling of connection with others’ stories, mutual learning among group members, digital format, and length of the workshop were features that the participants found particularly valuable.

Previous research has indicated that lack of language competence could be a barrier to minority groups’ community program engagement [36]. Some Korean mothers in our study expressed appreciation that their native language was used in the workshop, as limited English proficiency would have made it more difficult for them to express their emotions and thoughts. Having interpreters at our workshops and allowing participants to speak their native languages effectively addressed the potential language gaps and facilitated mothers’ active engagement throughout the workshop process. Furthermore, participants shared acculturation experiences, the experience of motherhood, and the responsibilities of taking care of their children may have enhanced the emotional resonance of the stories and the sense of mutual understanding.

Data drawn from the stories provided an in-depth understanding of the meaning-making, attitudes, perceptions, and cultural views about having their children vaccinated against HPV. The most common reason Vietnamese American and Korean American mothers offered for vaccinating their children was protection. Consistent with previous studies in these populations, our participants expressed a strong sense of autonomy with regard to vaccination decision-making and information seeking on HPV and the vaccine. Consistent with prior research [10,37], parents with higher awareness and knowledge about the benefits of the HPV vaccine and higher prioritization of protecting their children were more likely to vaccinate their children as a preventive measure.

Given the critical role health care providers play in providing evidence-based education about vaccination, a provider not recommending HPV vaccination during a clinical visit is a missed opportunity for a child to get timely protections [37]. Language barriers further hinder communication between immigrants and their providers. Prior studies have documented immigrants’ concerns about difficulties understanding English including medical jargon in clinic settings [38,39], highlighting how providing culturally and linguistically relevant health care service is essential. Health care providers, therefore, need to take the initiative and incorporate vaccine education in their practice to help parents and their children to make informed decisions, and, when needed, make an interpreter available.

Previous studies have demonstrated that immigrants rely on various sources of health information, including health care professionals, friends, family, and the internet [40,41]. Although Vietnamese American and Korean American mothers considered health professionals to be their primary source for HPV information, they also reported heavy reliance on the internet to find more information to check whether the information from other sources was reliable. The internet affords users immediate access to a wide variety of information and a variety of perspectives on the same topic with privacy, immediacy, convenience, and anonymity [42] and allows them to search for health information in their native language. However, health information on internet has also been criticized as a potential source of misinformation [43]. For example, some mothers expressed concerns about the prevalence of rumors and misinformation surrounding the HPV vaccine on social media platforms. This finding highlights the importance of educating Vietnamese American and Korean American mothers about reliable health care websites, as well as how to interpret and integrate information found on the internet with health information. Targeted educational interventions designed to increase health knowledge through the dissemination of culturally sensitive, relevant, and high-quality health information via web-based sources may also have great potential to make significant contributions to promoting HPV vaccinations for VAs and Korean Americans.

First-generation Vietnamese American and Korean American immigrant mothers can benefit from seeking support and help from different sources and personal networks to cope with feelings of inadequacy and frustration in adapting to everyday challenges in a new country, but especially the US health care system [40]. When immigrants enter a new cultural environment, building close relationships with home-nation friends in the host culture can help them obtain health information and support. Members of shared cultural social groups share similar experiences of living and studying in a new cultural environment and have many of the same concerns about dealing with various difficulties in their new countries. In our study, mothers expressed a desire to know about friends’ and family members’ experiences with HPV vaccination and discussed how receiving advice from family and friends influenced their decisions about their child’s HPV vaccinations, allowing for the possibility that conversations with, or recommendations from, friends and family members would help mothers become more familiar with benefits of the HPV vaccine and, in turn, be more likely to vaccinate their children against HPV.

### Limitations

Though our findings regarding feasibility and acceptability were encouraging and our qualitative analyses yielded numerous insights, these results may not generalize to other ethnic and racial populations. Also, recruiting busy mothers for 2-day workshops proved challenging in this study. Thus, the findings may be limited to mothers who were reachable, available at the time of the study, and had access to virtual videoconference. Future research could attempt a diverse recruitment approach to ensure a more representative sample.

### Conclusions

Our findings suggest that our novel DST workshops provided an easy-to-deliver, feasible, and acceptable method for engaging Vietnamese American and Korean American immigrant mothers from the community in the co-development culturally and linguistically congruent interventions. The findings also help us to better understand how factors such as spouse and partner opinions or insurance coverage influence Vietnamese American and Korean American immigrant mothers’ HPV vaccine decision-making.

## Abbreviations

DST: digital storytelling
HPV: human papillomavirus

## Data Availability

The data sets generated during and analyzed during this study are available from the corresponding author on reasonable request.
